# Anti-inflammatory Effects of Fungal Metabolites in Mouse Intestine as Revealed by *In vitro* Models

**DOI:** 10.3389/fphys.2017.00566

**Published:** 2017-08-07

**Authors:** Dominik Schreiber, Lisa Marx, Silke Felix, Jasmin Clasohm, Maximilian Weyland, Maximilian Schäfer, Markus Klotz, Rainer Lilischkis, Gerhard Erkel, Karl-Herbert Schäfer

**Affiliations:** ^1^Department of Biotechnology, University of Applied Sciences Kaiserslautern Kaiserslautern, Germany; ^2^Department of Biotechnology, Technical University of Kaiserslautern Kaiserslautern, Germany; ^3^Pediatric Surgery, University Hospital Mannheim Mannheim, Germany

**Keywords:** inflammatory bowel disease, mesenterial perfusion, galiellalactone, dehydrocurvularin, dexamethasone, ENS, neuroinflammation, intestine

## Abstract

Inflammatory bowel diseases (IBD), which include Crohn's disease and ulcerative colitis, are chronic inflammatory disorders that can affect the whole gastrointestinal tract or the colonic mucosal layer. Current therapies aiming to suppress the exaggerated immune response in IBD largely rely on compounds with non-satisfying effects or side-effects. Therefore, new therapeutical options are needed. In the present study, we investigated the anti-inflammatory effects of the fungal metabolites, galiellalactone, and dehydrocurvularin in both an *in vitro* intestinal inflammation model, as well as in isolated myenteric plexus and enterocyte cells. Administration of a pro-inflammatory cytokine mix through the mesenteric artery of intestinal segments caused an up-regulation of inflammatory marker genes. Treatment of the murine intestinal segments with galiellalactone or dehydrocurvularin by application through the mesenteric artery significantly prevented the expression of pro-inflammatory marker genes on the mRNA and the protein level. Comparable to the results in the perfused intestine model, treatment of primary enteric nervous system (ENS) cells from the murine intestine with the fungal compounds reduced expression of cytokines such as IL-6, TNF-α, IL-1β, and inflammatory enzymes such as COX-2 and iNOS on mRNA and protein levels. Similar anti-inflammatory effects of the fungal metabolites were observed in the human colorectal adenocarcinoma cell line DLD-1 after stimulation with IFN-γ (10 ng/ml), TNF-α (10 ng/ml), and IL-1β (5 ng/ml). Our results show that the mesenterially perfused intestine model provides a reliable tool for the screening of new therapeutics with limited amounts of test compounds. Furthermore, we could characterize the anti-inflammatory effects of two novel active compounds, galiellalactone, and dehydrocurvularin which are interesting candidates for studies with chronic animal models of IBD.

## Introduction

Inflammatory bowel diseases (IBD), such as Crohn's disease and ulcerative colitis are acute and chronic inflammatory conditions. Both conditions are characterized by an infiltration of mucosal macrophages and lymphocytes into the intestinal tissue producing a wide range of pro-inflammatory cytokines such as TNF-α, IL-1, IL-6, and IFN-γ which are directly implicated in the pathogenesis of the diseases (Neurath, 2014). In addition to mucosal immune and intestinal epithelial cells, neurons, and glial cells of the enteric nervous system (ENS) participate in the onset and progression of IBD. Under normal physiological conditions, the ENS actively controls intestinal motility, digestion, secretion and intestinal barrier functions (Keita and Soderholm, 2010). In patients with IBD, alterations of the ENS such as apoptosis of neurons and glial cells led to gut dismotility, secretion disorders, diarrhea and hypersensivity of the intestine (Lakhan and Kirchgessner, 2010; Margolis et al., 2011; Demir et al., 2013). In addition, activation of enteric glial cells and the concomitant induction of the expression of pro-inflammatory mediators (e.g., S100B, TNF-α, IL-1β, iNOS) promote chronic inflammation in the gut mucous layer by recruitment of immune cells (Capoccia et al., 2015).

Current therapies for IBD comprise glucocorticoids, biopharmaceuticals (e.g., targeting TNF-α), aminosalycilates and immunosuppressive drugs (e.g., methotrexate) (Bouguen et al., 2011; Burger and Travis, 2011; Mowat et al., 2011). Most of the available therapies for IBD do not even sufficiently retard disease progression or exhibit deleterious side effects, such as immuno-suppression (Bernstein, 2014; Neurath, 2014). Hence, there is a need to identify new pharmacological approaches and new drug targets in order to improve the current treatment regimens for IBD. Here, appropriate tools should be used to investigate the impact of these new compounds. Due to their specific advantages, *in vitro* models with intestinal segments are widely used for research in intestinal physiology and pharmacology. Recently, more complex *in vitro* perfusion approaches have been described by several groups for the analysis of drug-effects on motility (Lautenschlager et al., 2010; Alam et al., 2012; Schreiber et al., 2014). Due to the ENS's ability to control most intestinal functions autonomously, intestinal segments can keep some of their normal physiological functions *in vitro* without CNS support. Therefore isolated, perfused intestinal segments are also suitable models for the analysis of intestinal motility (Vantrappen et al., 1965; Benard et al., 1997; Ferens et al., 2005; Lammers, 2005; Seerden et al., 2005; Lammers and Cheng, 2008) as well as other physiological functions (Adachi et al., 2003; Song et al., 2006). In addition, effects of drugs on intestinal motility can also be recognized at a very early stage of development. Only very small amounts of an experimental compound are needed in comparison to classical animal experiments, where several milligrams of a compound are needed.

In the current study, we used an inducible *in vitro* model that combines both vascular and luminal perfusion of the mouse intestine. This model allows to investigate the impact of pro-inflammatory stimuli on the gut wall by perfusing the gut tissue with a pro-inflammatory cytokine mix (CM) for a defined range of time. The tissues can be harvested after induction of inflammation and analyzed in detail. Moreover, the model allows the online treatment with anti-inflammatory compounds, such as the fungal metabolites galiellalactone and dehydrocurvularin, or the well-established antiinflammatory compound dexamethasone. Galiellalactone and dehydrocurvularin are cyclic lactones, which have been shown to interfere with NF-κB, JAK/STAT, and TGF-β signaling (Weidler et al., 2000; Hellsten et al., 2011; Rudolph et al., 2012). In a previous study we demonstrated that the closely related macrocyclic lactone S-Curvularin which differs from dehydrocurvularin by the lack of the double bond at C-10 and C-11, reduced the expression of pro-inflammatory genes on mRNA and protein level in the model of collagen-induced arthritis in mice without obvious acute toxic effects (Schmidt et al., 2012). In addition, galiellalactone ameliorated inflammation and thrombosis in a model of Apolipoprotein E (ApoE)—deficient mice and reduced experimental asthma (Hausding et al., 2011; Bollmann et al., 2015). According to our previous data, dehydrocurvularin and galiellalctone seem to be promising new compounds for the treatment of chronic inflammatory diseases, but no data exist on their efficacy in a model IBD.

## Materials and methods

### Cell culture

The human colon adenocarcinoma cell line DLD-1 (DSMZ ACC278) was obtained from the German Collection of Microorganisms and Cell Cultures (DSMZ Braunschweig, Germany) and grown in RPMI 1640 medium containing 25 mM HEPES buffer and 2 mM L-glutamine, supplemented with 10% FCS, 100 units/ml penicillin and 100 μg/ml streptomycin at 37°C and 5% CO_2_. DLD-1 cells were seeded into 6-well plates at a cell density of 5 × 10^5^ cells/ml in RPMI 1640 medium with 10% FCS and allowed to grow for 24 h. After starving the cells for 16 h in RPMI 1640 medium containing 0.5% FCS, cells were pretreated with or without the test compounds for 1 h and induced with a cytokine mix (CM) consisting of the cytokines 10 ng/ml IFN-γ (recombinant, human, ImmunoTool, Germany), 10 ng/ml TNF-α (recombinant, human, ImmunoTool, Germany) and 5 ng/ml IL-1β (recombinant, human, ImmunoTool, Germany) for additional 5 h. Untreated samples and samples without stimulation served as controls.

### Animals

Adult BALB/c mice of both sexes, age 5 to 8 months were used for the perfusion experiments as well as for the isolation of ENS networks for cytokine release and qRT-PCR. Myenteric plexus cultures were set up from mice aged 1 to 5 days. Animal experiments were approved by German legislation and reported to the responsible authorities, the approval protocol number for the perfusion experiments was A 11-20-002 § 6. For ENS isolation, adult mice were killed by cervical dislocation. Postnatal animals were killed by decapitation. Intestinal resection for perfusion was performed as described under Intestinal Perfusion and Perfusion System. Animals which were sacrificed for ENS isolation were reported to the responsible authorities according to local laws.

**Figure 1:**
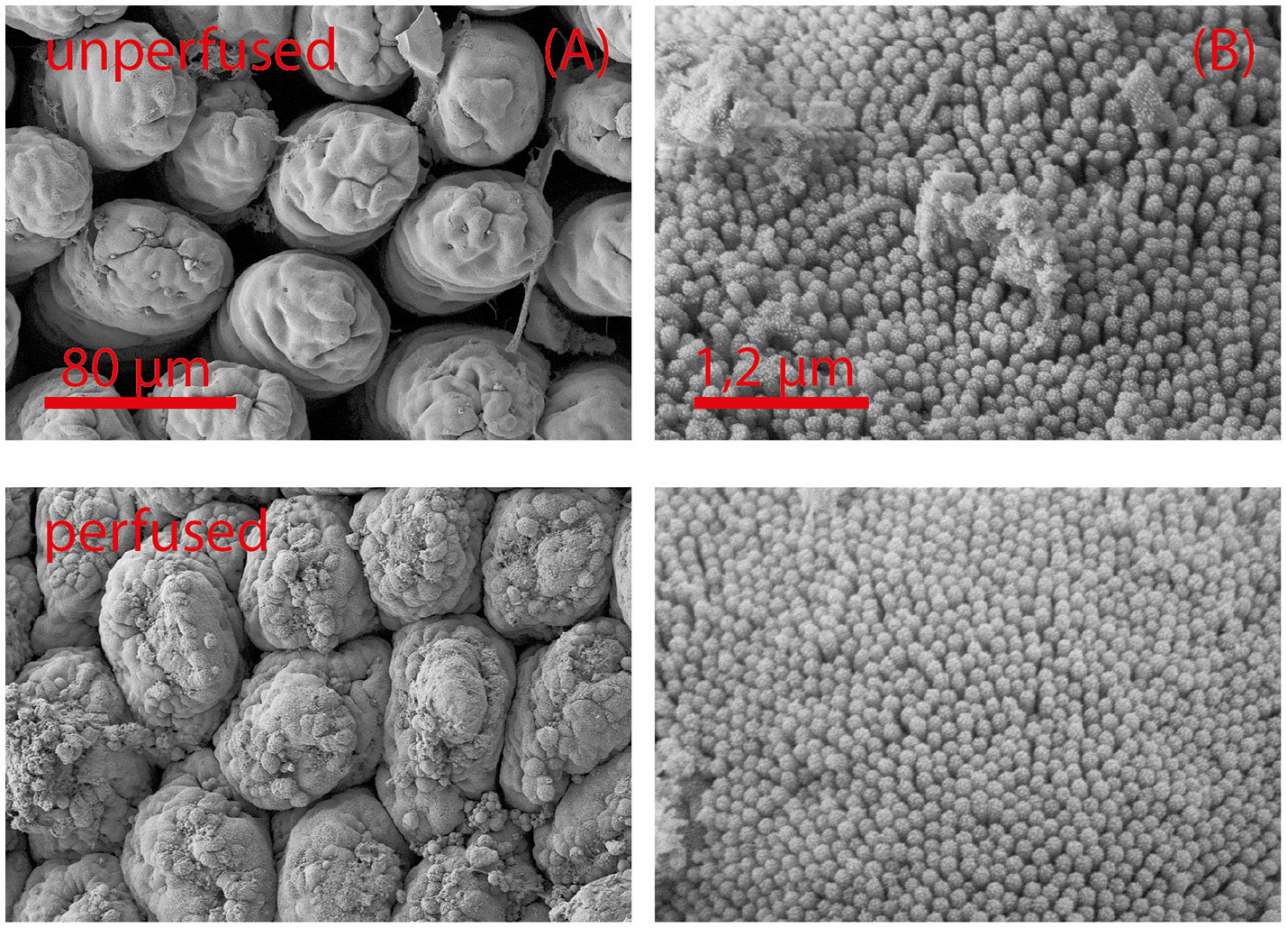
Scanning electron microscopy images **(A,B)** of unperfused intestinal tissue (upper row) and after 5 h of perfusion (lower row). Intestinal segments were perfused as described in the Materials and Methods section. Tissue damage is most pronounced at the tips of the mucosal villi **(A)**. Ultrastructural characteristics (microvilli) remain intact **(B)**. Images from the side of a villus are shown in **(B)**.

**Figure 2:**
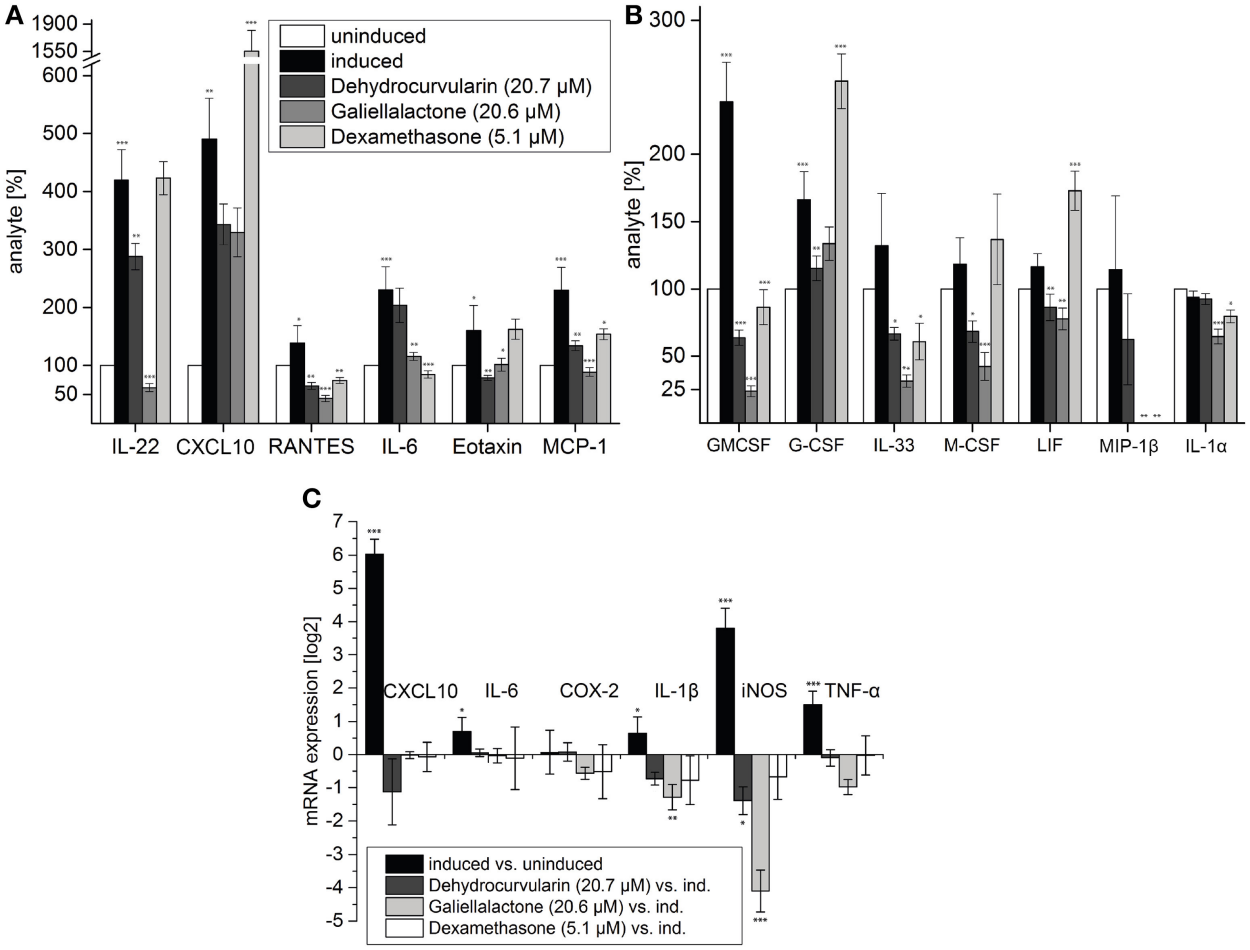
Multiplex ELISA of tissue lysates **(A,B)** and qRT-PCR **(C)** of pro-inflammatory transcripts in perfused, intestinal tissue samples after 5 h of perfusion. During the first hour of perfusion, the indicated concentrations of test compounds were added to the perfusion medium. During the following 4 h, a CM-stimulus was added in addition. Multiplex ELISA: Tissue samples of five (induced, uninduced, dehydrocurvularin treated) or four (galiellalactone and dexamethasone treated) independent experiments were measured twice. The cytokines CXCL10, RANTES, IL-6, Eotaxin, and MCP-1 which are also regulated in adult ENS networks (Figure 3) are shown in **(A)**. Further regulated cytokines are shown in **(B)**. The data are presented in comparison to uninduced control tissue (±*SD*). The uninduced control tissue corresponds to 100%. ^*^p < 0.05; ^**^p < 0.01; ^***^p < 0.001 treated tissue vs. cytokine induced tissue. qRT-PCR: Induced vs. uninduced samples and treated vs. induced samples are shown. Results were normalized to GAPDH, *n* = 5. The data are presented as log2 ratios in comparison to cytokine induced tissue (±*SD*). ^*^p < 0.05; ^**^p < 0.01; ^***^p < 0.001 treated tissue vs. cytokine induced tissue.

For intestinal perfusion experiments, data from 25 animals is shown. From one perfused intestine, tissue for Multiplex ELISA measurements, qRT-PCR and histology could be resected (Figures 1, 2).

For the experiments with ENS networks from adult mice, 25 animals were used. From the ENS cultures, cells for qRT-PCR and supernatants for Multiplex ELISA could be obtained.

In addition, results obtained with ENS networks from 5 animals are shown in **Figure 4**.

For the experiments with postnatal ENS single cells, cells were pooled before seeding them in 24 well plates. A total number of 39 animals were used for this type of experiments (**Figure 5** and Supplementary Figure 3).

That is a total number of 94 animals used for the results shown in this manuscript.

### Intestinal perfusion and perfusion system

Intestinal resection for perfusion was performed under Ketamine/Xylazine/Acepromazine (100, 15, 3 mg/kg, intraperitoneal injection) anesthesia. The mesenteric artery was cannulated with a BD Vasculon Plus 26GA × 19 mm intravenous catheter and all blood vessels leading to a 2.5 to 3 cm proximal jejunal segment were spared, while all remaining vessels and gut tissues were ligated and dissected. The resection procedure is described in Supplementary Figure 1A. After cannulation, the gut was immediately flushed with perfusion medium through the artery and placed in the organ bath chamber.

Perfusion was performed both luminally (0.2 ml/min) and through the mesenteric artery (0.1 ml/min) with an REGLO Digital MS-4/12 pump (Ismatec, Germany). Anti-inflammatory compounds and cytomix was added mesenterially. The custom designed perfusion chamber (6.7 × 5 × 30 cm) was flushed with Tyrode solution, that superfused the jejunal segment at a flow rate of about 100 ml/min solution (Lammers, 2005). Only jejunal segments from the small intestine were used in the perfusion study. The Tyrode solution in the perfusion bath was gassed with carbogen (95% O_2_, 5% CO_2_) prior to perfusion. The pH and temperature were constant at 7.35 ± 0.05 and 37.5 ± 0.1°C, respectively. A scheme of the perfusion setup is depicted in Supplementary Figure 1B. For blood vessel perfusion and flushing of the intestinal lumen, a tissue culture medium was used. The contents of the perfusion medium are listed in Table 1. The osmolarity of the perfusion medium was 302 mosm/kg. A modified DMEM/F12 without phenol red and with 25 mM HEPES was used (P04-41659, PAN-Biotech, Germany). Changes to the original medium are listed in Table 1. The pH of this medium after gassing with carbogen (5% CO_2_, 95% O_2_) was 7.35. The medium was diluted 4 parts with 1 part of FCS (Sera Plus, PAN-Biotech, Germany). 100 μg/ml penicillin (Penicillin G-Na, Applichem, Germany), 100 μg/ml streptomycin (Streptomycinsulfate, Applichem, Germany), 70 pg/ml norepinephrine (Arterenol, Sanofi, Germany) and 9 milliU/l insulin (Insuman Rapid, Sanofi, Germany) were added before the perfusion experiment.

**Table 1:** Contents of the mesenterial perfusion medium.

**MODIFIED FINAL CONCENTRATION (MG/L)**	
Sodium hydrogen carbonate	1400.0000
Glucose	900.0000
L-glutamine	131.0000
Sodium pyruvate	25.0000
DL-68-Lipoic acid	0.0470
**SUPPLEMENTS (MG/L)**	
BSA	30000.0000
α-Tocopherol	20.0000
Natriumascorbat	15.0000
Transferrin	5.0000
Catalase	2.5000
SOD	2.5000
Glutathione (reduced)	1.0000
Selenium	0.1900
Cortisol	0.1000
Retinol (acetate)	0.1000
Retinol (all trans R.)	0.1000
Ergocalciferol	0.0600
Triiodothyronine	0.0015

Perfused segments were pretreated with test compounds (dissolved in ethanol, final concentration of ethanol in the culture medium 0.1%), or with vehicle (0.1% of EtOH, uninduced and induced control only) for 1 h and subsequently perfused with either the test compounds (dissolved in ethanol, final concentration of ethanol in the culture medium 0.1%) and cytokines or the vehicle and cytokines (induced control) or vehicle only (uninduced control) for 4 h. During pretreatment no cytokines were added to the perfusion medium. As an inflammatory stimulus, a cytokine mix (CM) (Schmidt et al., 2012) consisting of 10 ng/ml IFN-γ (recombinant, murine, ImmunoTool, Germany), 10 ng/ml TNF-α (recombinant, murine, ImmunoTool, Germany) and 5 ng/ml IL-1β (recombinant, murine, ImmunoTool, Germany) was used. After perfusion, tissue samples (20 mg) were resected for RNA isolation and Multiplex ELISA.

Long-time motility heatmaps were generated as described previously by Schreiber et al. (2014) only to evaluate the quality of the perfusion. Motility could be kept constant for at least 5 h.

### Myenteric plexus cultures

Myenteric plexus from adult and postnatal BALB/c mice were obtained by digestion with LiberaseTH (Roche, Germany) and subsequent mechanic treatment, as previously described (Grundmann et al., 2015). Cultures (postnatal) and intact myenteric networks (adult) were used to investigate either iNOS expression or cytokine release and expression. Postnatal myenteric plexus was dissociated and plated on ECM (Sigma-Aldrich, Germany) coated coverslips. The cells were cultivated in DMEM/F12 medium (Life Technologies, Germany) with 1% penicillin/streptomycin solution 100x (PAN Biotech, Germany), 1% BSA (35%) solution (Sigma-Aldrich, Germany), 2% B27 with retinoic acid (PAA Laboratories, Germany) and 100 ng/ml GDNF (recombinant, human, ImmunoTools, Germany).

Adult myenteric plexus networks were kept in 24 well plates for 24 h, prior to collecting the supernatant or the tissue for PCR analysis. Here Neurobasal medium P was used instead of DMEM/F12. Cytomix and anti-inflammatory compounds were added in the culture medium at appropriate time points.

### Test compounds

Dehydrocurvularin and galiellalactone were obtained by fermentation of the producer strains *Penicillium* sp. IBWF3-93 and the ascomycete IBWFA111-95, respectively. Isolation of the compounds from the culture fluid by extraction with ethyl acetate and preparative HPLC was performed as described previously (Weidler et al., 2000; Rudolph et al., 2012). The purity of the compounds was >98% as determined by HPLC equipped with diode-array detection and mass spectrometry analysis. Dexamethasone was obtained from Applichem (Germany).

In initial studies, the concentrations of each compound were chosen according to earlier studies. 10.3 μM (2 μg/ml) galiellalactone, 10.3 μM (3 μg/ml) dehydrocurvularin, 2.5 μM (1 μg/ml) dexamethasone which were used on ENS cells were tested in the perfusion model with little to no effect (data not shown). Therefore, higher compound concentrations were used for intestinal perfusion. In the perfusion model 20.7 μM (4 μg/ml) dehydrocurvularin, 20.6 μM (6 μg/ml) galiellalactone and 5.1 μM (2 μg/ml) dexamethasone were used. Due to the complexity of organ resection and perfusion, only one concentration of every compound was evaluated in this model.

### RNA isolation

Intestinal tissue samples were stored in RNAlater (Ambion, USA) at −80°C. Total RNA of intestinal segments was prepared by homogenizing the samples in lysis buffer (buffer RLT, Qiagen, Germany). ENS networks and DLD-1 cells were collected after treatment, washed with PBS and treated likewise. Total RNA was prepared from intestinal tissue lysates, ENS network lysates and DLD-1 cells using the RNeasy Mini Kit (Qiagen, Germany) according to the manufacturer's instructions. RNA was transcribed to cDNA directly after isolation with a RevertAid H Minus First Strand cDNA synthesis kit (Thermo Scientific, Germany) according to the manufacturer's instructions. For reverse transcription, 1 μg of RNA from intestinal samples and DLD-1 cells and 100 ng of RNA from ENS samples were used. RNA and cDNA samples were stored at −80°C.

### Quantitative real-time polymerase chain reaction analysis (qRT-PCR analysis)

To analyze mRNA expression RT and qPCR analysis was done as described before (Jung et al., 2009). qRT-PCR analysis was performed in a total volume of 20 μl in 96-well plates using a CFX96 Real-Time PCR System (Bio-Rad, USA). For real-time PCR (initial heating step 15 min at 95°C, 40 cycles of 15 s at 94°C, 30 s at 56°C and 30 s at 72°C), the following mouse specific sense and antisense primer were used for mRNA expression profiling in mouse cells and tissues: COX-2 sense CTCTACATAAGCCAGTGAGA, antisense TCATCTTGTAACAACACTCAC; GAPDH sense ACCCAGAAGACTGTGGATGG, antisense ACACATTGGGGGTAGGAACA; IL-1β sense AAGGAGAACCAAGCAACGACAAAA, antisense TGGGGAACTCTGCAGACTCAAACT; IL-6 sense CTGCAAGAGACTTCCATCCAGTT, antisense GAAGTAGGGAAGGCCGTGG; iNOS sense GGCAGCCTGTGAGACCTTTG, antisense GCATTGGAAGTGAAGCGTTTC; CXCL10 sense TCCTTGTCCTCCCTAGCTCA, antisense ATAACCCCTTGGGAAGATGG; TNF-α sense CCAGTGTGGGAAGCTGTCTT, antisense AAGCAAAAGAGGAGGCAACA. The following human specific primers were used for mRNA expression profiling in DLD-1 cells: COX-2 sense TTCAAATGAGATTGTGGGAAAATTGCT, antisense AGATCATCTCTGCCTGAGTATCTT; CXCL10 sense TGAGCCTACAGCAGAGGAA, antisense TACTCCTTGAATGCCACTTAGA; GAPDH sense CCTCCGGGAAACTGTGG, antisense AGTGGGGACACGGAAG; iNOS sense GCAGGTCACTTATGTCACTTATC, antisense GTTCTCAAGGCACAGGTCTC; IL-1β sense AAGCTGAGGAAGATGCTG, antisense ATCTACACTCTCCAGCTG; IL-8 sense TGCCAAGGAGTGCTAAAG, antisense CTCCACAACCCTCTGCAC; TNF-α sense TCTTCTGCCTGCTGCACTTTGG, antisense ATCTCTCAGCTCCACGCCATTG. To calculate the relative mRNA expression the method proposed by Pfaffl (2001) was used.

### Cytokine quantification: multiplex elisa and proteome profiler

In order to quantify cytokine concentrations in perfused tissues or cell culture supernatants after inflammatory stimulation or anti-inflammatory treatment, Multiplex ELISA or proteome profiler assays were used.

Multiplex ELISA experiments were performed with a Milliplex MAP Mouse Cytokine/Chemokine Magnetic Bead Panel Immunoassay Kit (Merck Millipore, Germany) according to the manufacturer's instructions on a Bio-Rad Bio-Plex 200 System. Protein concentration was measured with a Pierce BCA Protein Assay (Thermo Scientific, Germany) or a Qubit Protein Assay Kit (ThermoFisher Scientific, Germany) according to the manufacturer's instructions. Tissue lysates were generated from tissue samples resected after perfusion. Tissue samples were instantly frozen at −80°C after the perfusion experiment, freeze dried and lysed with a bead mill. ENS culture supernatants from adult ENS networks were centrifuged at 1,000 × g for 5 min and frozen at −80°C. Results were normalized to the protein content and expressed relative to the uninduced control of the respective tissue. Cytokine contents of the cell culture supernatant were normalized to the protein content of the cells and expressed likewise.

For analyses of cytokine and chemokine production in culture supernatants of DLD-1 cells, the “Human Cytokine Array Panel A” array system (R&D Systems, Germany) was used according to the manufacturer's instructions. ImageJ software was used for the quantification of the protein spots. DLD-1 cells were starved for 16 h in RPMI 1640 medium with 0.5% fetal calf serum. The cells were seeded at 5 × 10^6^ cells/ml in 6-well plates, pretreated with or without test compound for 1 h and induced with a cytokine mix (CM) for additional 16 h as described in Section Cell Culture.

### Immunohistochemisty

ENS single cells were fixated with 4% formaldehyde in PBS at 5°C for 17 min on glas coverslips. Adult ENS networks were centrifuged, washed with PBS and separated from PBS residues. They were embedded in Tissue-Tek by freezing at −80°C. Tissue-Tek slices (10 μM) were made with a Kryostat and fixated with ice cold Acetone 70% (Applichem, 70% + 30% water). As primary antibodies rabbit anti iNOS (polyclonal, ab15323, Abcam), rabbit anti GFAP (polyclonal, Z0334, Dako, Germany) and mouse anti Tubulin β III (monoclonal, MAB1637, Merck Millipore, Germany) were used. Alexa Fluor 594 goat anti rabbit (Invitrogen, USA) and goat anti mouse 488 (Invitrogen, USA) were used as secondary antibodies. Rabbit IgG (Dako, Germany) was used for the isotype control. 4′,6-diamino-2-phenylindole (DAPI, Sigma-Aldrich, Germany) was used for nuclear stainings.

### Scanning electron microscopy

Intestinal tissue samples were fixated in 100 mM cacodylate buffer with 2.5% glutaraldehyde for 24 h at room temperature. After osmium tetroxide treatment and dehydration with an increasing ethanol concentration and HMDS, they were sputtered with gold. Standard scanning electron microscopy imaging on the samples was carried out on a Supra40 by ZEISS (Germany) in low kV mode at 2.5 kV and 5.3 mm working distance.

### Statistical methods

For statistical analysis, a one-way ANOVA followed by Tukey's multiple comparison test for the comparison of multiple means was used. In order to test if the inflammatory induction in the positive control samples of qRT-PCR experiments was significantly >0, a one sample *t*-test was used. Data with a *p* < 0.05 was regarded as statistically significant. Data was marked with ^*^ for *p* < 0.05, ^**^ for *p* < 0.01 and ^***^ for *p* < 0.001. Results are presented as mean ± *SD* wherever possible.

## Results

### Effect of dehydrocurvularin and galiellalactone on the expression of pro-inflammatory marker genes in perfused intestinal segments

Using specifically adapted perfusion media avoided capillary leaking and edema. Combined with a continuous oxygenation good tissue preservation could be maintained (Figure 1). After 5 h of perfusion, only minor damages in the mucosal structure at the tips of the villi could be observed. These damages did not interfere with gut motility. The motility pattern stayed constant even during extended perfusion periods (Supplementary Figure 2). Perfusion with a mixture of pro-inflammatory cytokines (CM) did not alter tissue integrity.

To test the effects of dehydrocurvularin, galiellalactone, or dexamethasone treatment in the setting of chronic inflammatory bowel disease, we studied the expression profile of pro-inflammatory marker genes by real time PCR in perfused intestinal segments that were either perfused with vehicle (0.1% EtOH) alone or test compounds stimulated with a mixture of pro-inflammatory cytokines (CM) and identical vehicle-concentrations. Values derived from the homogenized tissues were expressed as ratios (log2) of relative mRNA content of CM induced vs. unstimulated tissue or vs. tissue which was treated with the individual compounds and with cytokines. The values were based on glyceraldehyde-3-phosphate dehydrogenase (*gapdh*) references that were determined in the same sample. Administration of CM through the mesenteric artery caused a significant up-regulation of mRNA levels for CXCL10 (64-fold), iNOS (14-fold), and TNF-α (3-fold) whereas IL-1β mRNA-level was only slightly induced (Figure 2C). Treatment with dehydrocurvularin (20.7 μM) or galiellalactone (20.6 μM) significantly down-regulated the mRNA levels of iNOS. For IL-1β mRNA expression, only galiellalactone lead to a significant decrease. In addition, a reduction of COX-2 and TNF-α mRNA expression by galiellalactone could be observed whereas CXCL10 mRNA levels were inhibited by dehydrocurvularin (Figure 2C).

To analyze the effects of dehydrocurvularin and galiellalactone on a broader range of cytokines and chemokines, we performed a Multiplex ELISA analysis with a mouse cytokine/chemokine immunoassay kit (Merck Millipore). For these experiments we used total protein lysates from perfused unstimulated and CM-stimulated intestinal segments treated with test compounds as described in the Materials and Methods Section (Intestinal perfusion and perfusion system). CM-treatment significantly elevated the expression of chemokines such as CXCL10, Eotaxin, MCP-1 (CCL2), and RANTES (CCL5) which are responsible for the recruitment of immune cells to the inflamed tissue (Figure 2B). Treatment of the tissue with galiellalactone or dehydrocurvularin significantly reduced the amount of most cytokines and chemokines. The amounts of the chemokines CXCL10, RANTES, MCP-1, LIF, and MIP-1β were decreased after anti-inflammatory treatment with dehydrocurvularin or galiellalactone. An increase in cytokine or chemokine secretion after treatment with the experimental compounds could not be observed. In contrast to the fungal lactones, dexamethasone significantly increased CXCL10, G-CSF, and LIF expression compared to the CM-induced control. Besides chemokines, the expression of pro-inflammatory, IBD-associated cytokines such as G-CSF, GM-CSF, M-CSF, IL-6, IL-22, and IL-33 was up-regulated upon vascular CM stimulation. Galiellalactone, dehydrocurvularin, and dexamethasone markedly inhibited CM-induced GM-CSF, IL-33, MCP-1, and RANTES expression. Dexamethasone treatment showed little effect on IL-22, Eotaxin, and M-CSF expression. In contrast, both new compounds decreased their expression. In summary, both fungal compounds displayed more pronounced anti-inflammatory effects than dexamethasone (5.1 μM) at the concentrations of 20.6 galiellalactone, respectively 20.7 μM dehydrocurvularin.

### Galiellalactone and dehydrocurvularin decrease pro-inflammatory marker gene expression in isolated adult ENS networks

**Figure 3:**
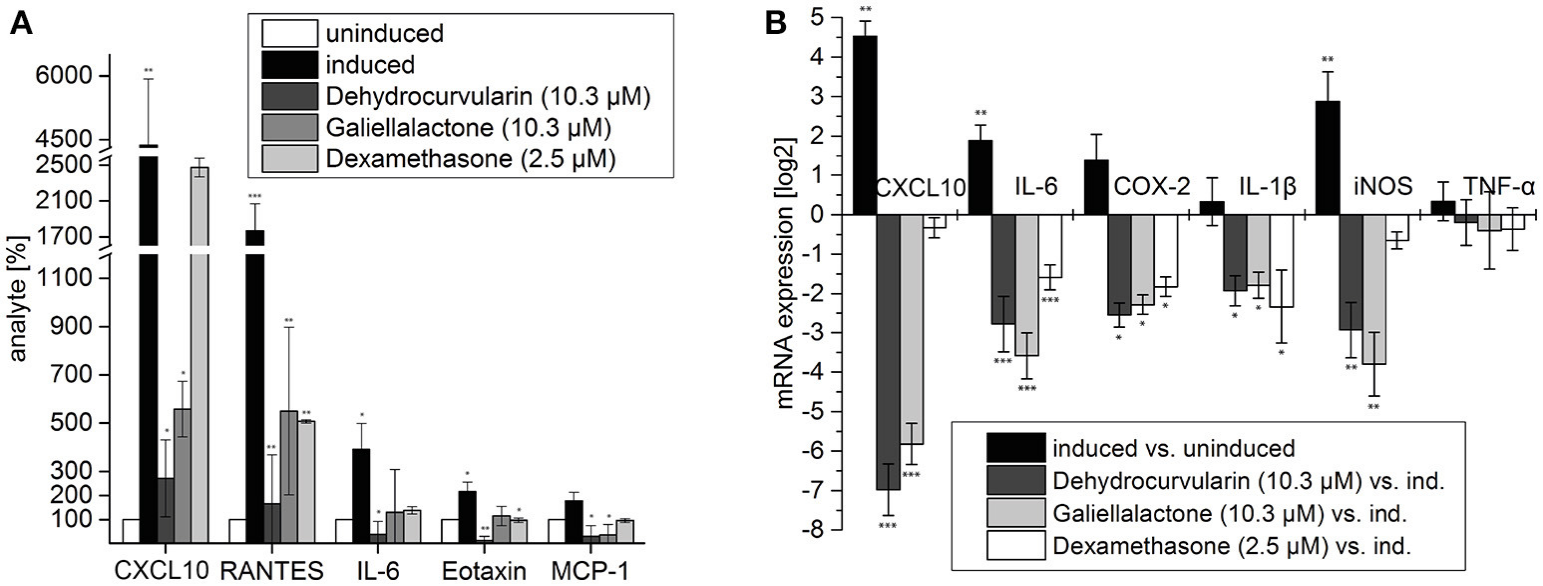
Multiplex ELISA of culture supernatants from adult ENS networks **(A)** and qRT-PCR analysis of pro-inflammatory transcripts in adult ENS networks **(B)** after treatment with the anti-inflammatory compounds galiellalactone and dehydrocurvularin. Cells were pretreated for 1 h with the indicated concentrations of test compounds prior to stimulation with CM for 24 h. Multiplex ELISA: The data are presented in comparison to untreated control cells (±*SD*). The untreated control cells correspond to 100%. *n* = 3 for the test substances galiellalactone and dehydrocurvularin, *n* = 2 for the positive control dexamethasone. ^*^p < 0.05; ^**^p < 0.01; ^***^p < 0.001 treated cells vs. cytokine induced cells. qRT-PCR: Induced vs. uninduced samples and treated vs. induced samples are shown. Results were normalized to GAPDH, *n* = 5. The data are presented as log2 ratios in comparison with cytokine induced control cells (±*SD*). ^*^p < 0.05; ^**^p < 0.01; ^***^p < 0.001 treated tissue vs. cytokine induced cells.

Stimulation of ENS networks with CM for 24 h caused a significant up-regulation of mRNA levels for CXCL10 (~22-fold), iNOS (~7-fold) and IL-6 (~3.5-fold). IL-1β as well as TNF-α and COX-2 were only slightly induced by the cytokine stimulus. Galiellalactone (10.3 μM) and dehydrocurvularin (10.3 μM) significantly down-regulated the mRNA levels of all transcripts analyzed except TNF-α, whereas dexamethasone (2.5 μM) did not significantly affect CXCL10, iNOS, and TNF-α mRNA levels (Figure 3B).

To analyze the effects of the fungal compounds on the CM-inducible synthesis of inflammation-related proteins, we performed a Multiplex ELISA analysis for pro-inflammatory mediators using cell culture supernatants of isolated adult ENS networks (Figure 3A). Treatment of ENS networks with CM for 24 h significantly elevated the expression of the chemokines CXCL10 (~44-fold), RANTES (~18-fold), Eotaxin (~2-fold), MCP-1 (~2-fold), and the cytokine IL-6 (~4-fold). The test compounds reduced the expression of these proteins albeit with different efficacies. Dehydrocurvularin (10.3 μM) and galiellalactone (10.3 μM) strongly inhibited the inducible expression of all investigated inflammation-related proteins. For the pro-inflammatory cytokines IL-6, Eotaxin, and MCP-1 cytokine levels were reduced below the level of the uninduced control by dehydrocurvularin. Supplementary data for the analyte IL-22 which responds to the treatment similar can be seen in Supplementary Figure 4. Dehydrocurvularin was more potent than galiellalactone on all cytokines which were tested. The effects of galiellalactone were at least comparable to dexamethasone at the concentrations used.

**Figure 4:**
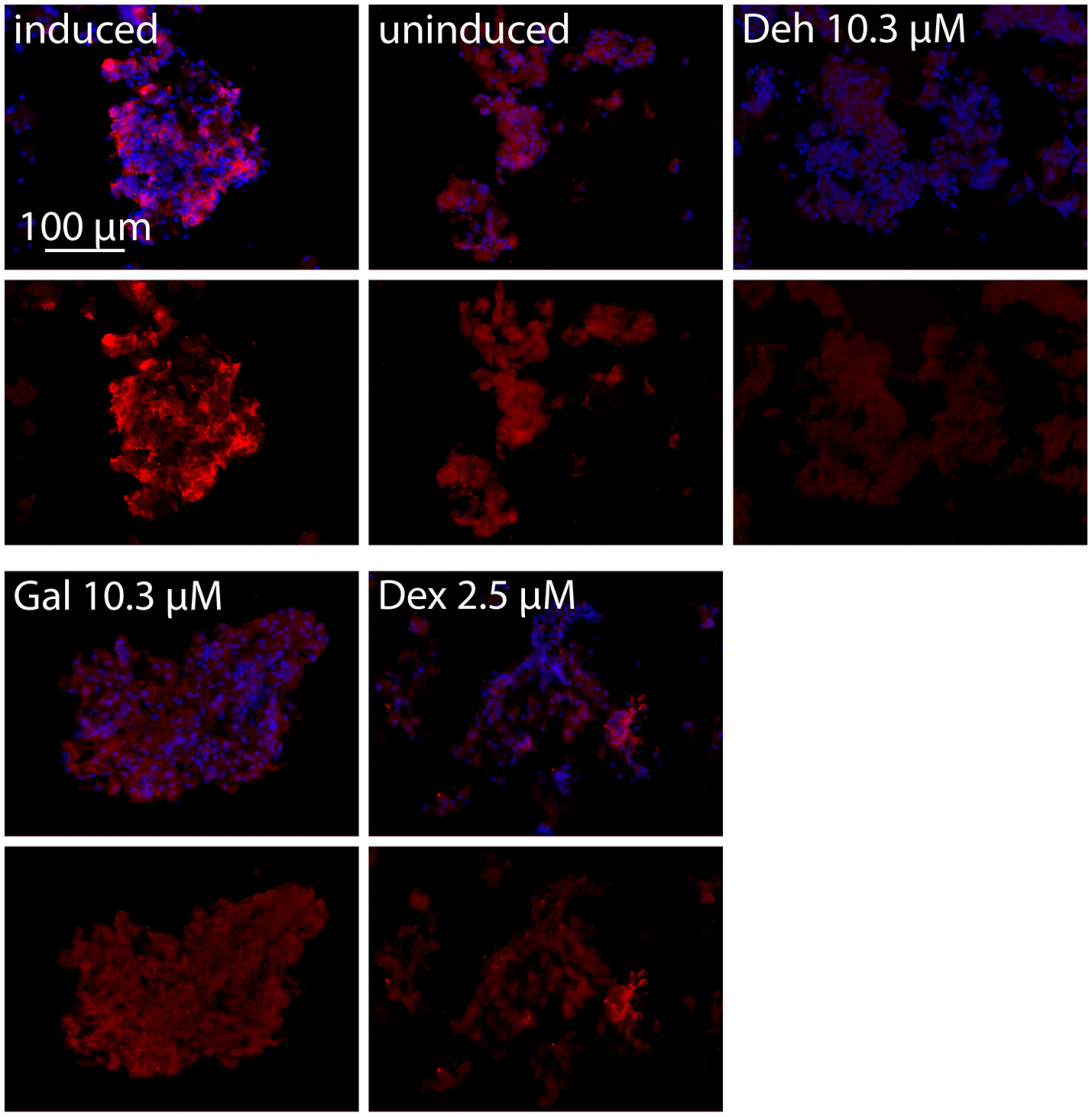
iNOS immunofluorescence staining of adult ENS networks after anti-inflammatory treatment. Cells were pretreated for 1 h with the indicated concentrations of test compounds prior to stimulation with CM for 24 h. iNOS positive cells (red, iNOS) and nuclei (blue, DAPI). Merge image (upper row), iNOS (lower row). Induced, cytokine induced culture; uninduced, uninduced control culture; Deh 10.3 μM, dehydrocurvularin 10.3 μM; Gal 10.3 μM, galiellalactone 10.3 μM; Dex 2.5 μM, dexamethasone 2.5 μM.

We further analyzed the effects of dehydrocurvularin, galiellalactone, and dexamethasone on iNOS expression in adult ENS networks by immunofluescence stainings (Figure 4). In comparison to the uninduced control, CM treatment strongly induced iNOS expression in ENS networks. In accordance with the qRT-PCR experiments, treatment of the cultured ENS cells with the test compounds resulted in a strong reduction of the iNOS staining. Application of dexamethasone (2.5 μM) also led to a decrease in iNOS expression.

### Effect of galiellalactone and dehydrocurvularin on iNOS expression in cultured postnatal ENS cells

**Figure 5:**
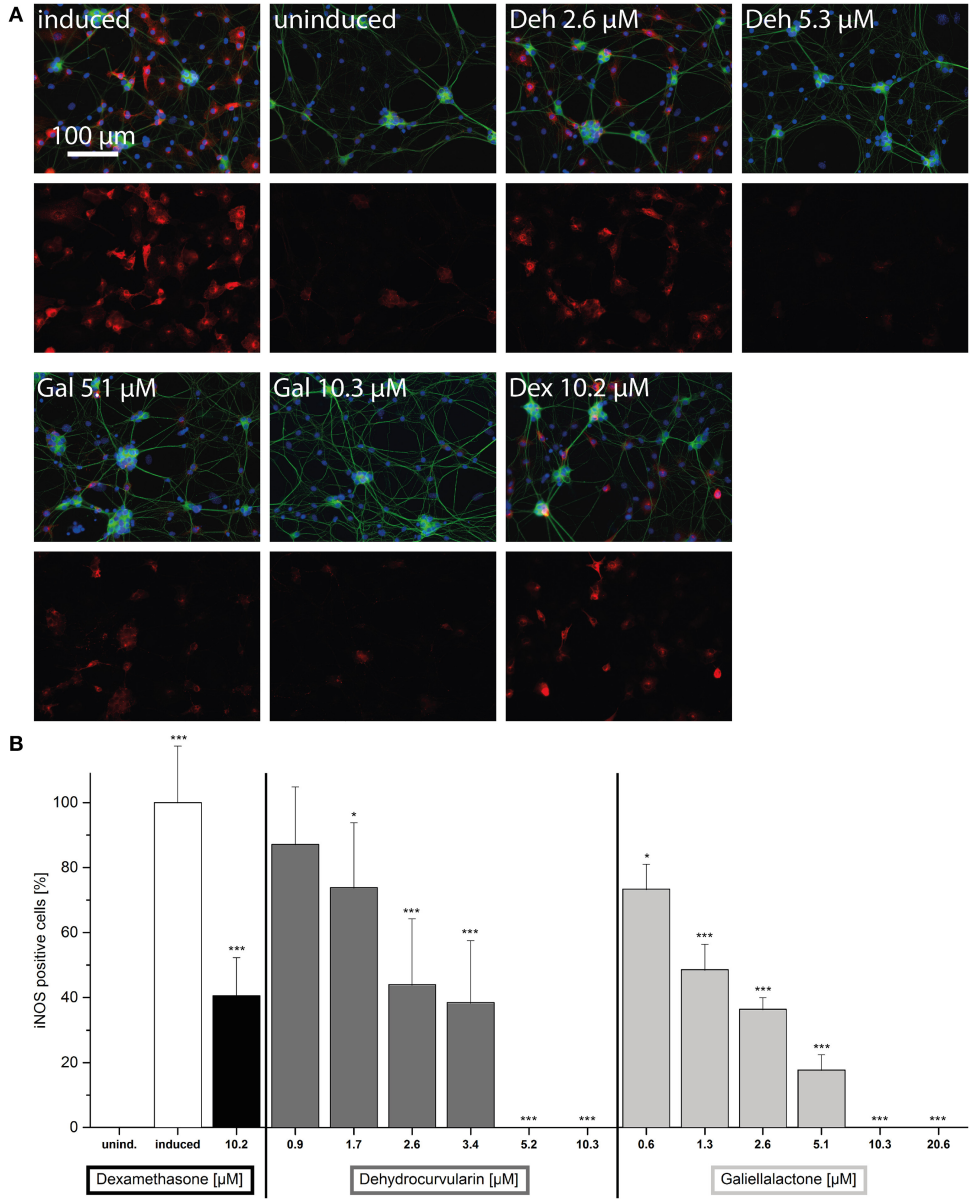
Effects of the anti-inflammatory compounds dehydrocurvularin, galiellalactone, and dexamethasone on iNOS expression in the postnatal ENS single cell culture. Cells were pretreated for 1 h with the indicated concentrations of test compounds prior to stimulation with CM for 24 h. **(A)** Neurons (green, βIII-Tubulin), iNOS positive cells (red, iNOS) and nuclei (blue, DAPI). The upper row shows a merge of all three fluorescence channels, the lower row the iNOS expression. (induced): cytokine induced culture, (uninduced): uninduced control culture, (Deh 2.6 μM): dehydrocurvularin 2.6 μM, (Deh 5.3 μM): dehydrocurvularin 5.3 μM, (Gal 5.1 μM): galiellalactone 5.1 μM, (Gal 10.3 μM): galiellalactone 10.3 μM, (Dex 10.2 μM): dexamethasone 10.2 μM. 5000 cells / coverslip. **(B)** Results are relative to positive control. The data are presented in comparison to cytokine induced control cells (±*SD*). ^*^p < 0.05; ^**^p < 0.01; ^***^p < 0.001, treated cells vs. cytokine induced cells, respectively cytokine induced cells vs. uninduced cells. At least 4 independent experiments were performed.

In order to quantify the effects of the test compounds on iNOS expression, immunofluorescence stainings of dissociated ENS single cells were performed. In this experimental setting, iNOS positive cells can be quantified by counting. After *in vitro* treatment of the cell cultures with CM for 24 h, two populations of cells were found: one highly iNOS positive, inducible with the CM stimulus population and the other iNOS negativ population (Figure 5A and Supplementary Figure 3). iNOS positive cells were of glial morphology. In the CM-treated cultures 21.0 ± 1.2% (*n* = 38) of all cells were iNOS positive, while in the uninduced culture not a single iNOS positive cell could be detected. Treatment with galiellalactone (10.3 μM) and dehydrocurvularin (5.3 μM) decreased the number of iNOS positive cells to the level of the uninduced control (Figures 5A,B) whereas dexamethasone (10.2 μM) only partially inhibited iNOS expression even at the highest concentration tested to around 50%.

Increasing concentrations of dehydrocurvularin or galiellalactone dose-dependently down-regulated iNOS expression in CM-treated postnatal ENS single cell cultures with IC_50_-values of 2.4 and 1.3 μM, respectively (Figure 5B). Complete inhibition of iNOS expression could be observed at 10.3 μM galiellalactone and 5.2 μM dehydrocurvularin. An overview staining of the postnatal ENS single cell culture confirmed that the culture consisted of neuronal and glial cells and only glial cells were iNOS positive as shown in Supplementary Figure 3.

### Galiellalactone and dehydrocurvularin decrease pro-inflammatory gene expression in DLD-1 cells

**Figure 6:**
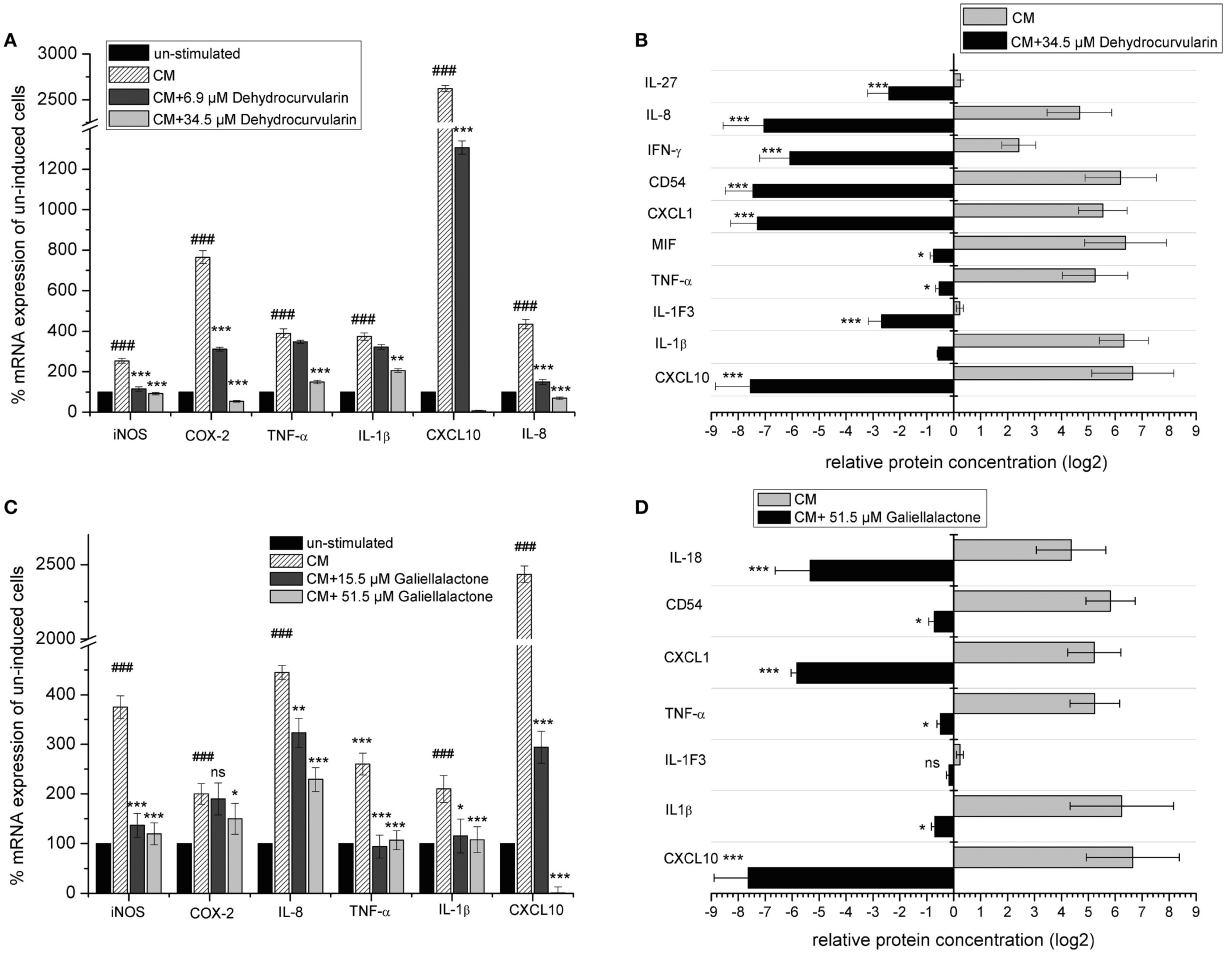
Effect of dehydrocurvularin and galiellalactone on mRNA levels and synthesis of pro-inflammatory genes in CM (TNF-α/IFN-γ/IL-1β) treated DLD-1 cells. **(A,C)** DLD-1 cells were pretretated with the indicated concentrations of test compounds before cytokine stimulation for 5 h. Total RNA was reversely transcribed and cDNAs measured as described in the Materials and Methods section. Values were expressed as relative mRNA content of CM induced vs. unstimulated cells [(###: *p* < 0.001 vs. unstimulated cells) and CM stimulated and compound treated cells vs. untreated, stimulated cells, corrected for *gapdh* as reference determined in the same sample in parallel. Data are shown as mean values ± *SD* of three independent experiments (^***^p < 0.001; ^*^p < 0.05 vs. stimulated cells; ns: not significant vs. stimulated cells)]. **(B,D)** Effect of galiellalactone and dehydrocurvularin on CM induced synthesis of pro-inflammatory proteins. DLD-1 cells were pretreated for 1 h with the indicated concentrations of test compounds prior to stimulation with CM for 16 h. The production of pro-inflammatory proteins in cell supernatants was determined with the human Cytokine Array (A) (R&D systems) according to the manufacturer's instructions. The analysis of two biological replicates (each one with two technical replicates) with ImageJ is shown. The data (means ± *SD*) represent relative protein amounts of significantly (>2-fold) regulated inflammation-related proteins (^***^p < 0.001; ^**^p < 0.01; ^*^p < 0.05 vs. stimulated cells; ns: not significant vs. stimulated cells).

The stimulation of DLD-1 cells with CM led to a significant up-regulation of mRNA levels for CXCL10, iNOS, IL-8, COX-2, IL-1β as well as TNF-α (Figures 6A,C). Both compounds strongly down-regulated the mRNA levels of all analyzed transcripts which was comparable to the results obtained with isolated adult and postnatal ENS cells. We also investigated the influence of dehydrocurvularin and galiellalactone on the inducible synthesis and secretion of pro-inflammatory cytokines and chemokines in the human DLD-1 cell line with an antibody array. After stimulation of the cells with CM, dehydrocurvularin almost reduced the expression of the chemokines CXCL-10, CXCL-1, and IL-8 as well as the cell surface glycoprotein ICAM-1 (CD54) to the level of unstimulated control cells (Figures 6B,D). The expression of the cytokines IL-27, IFN-γ, and IL-1F3 (IL-1ra) was strongly reduced after dehydrocurvularin treatment (>70%), whereas the effects on TNF-α, IL-1β, and MIF expression were less pronounced (Figure 6B). Galiellalactone strongly inhibited the inducible expression of IL-18, CXCL-1, and CXCL-10 (>90%) whereas the inducible expression of the pro-inflammatory cytokines TNF-α and IL-1 was not significantly affected (Figure 6D).

In summary, our data demonstrate that the fungal lactones dehydrocurvularin and galiellalactone are potent inhibitors of pro-inflammatory gene expression in *in vitro* models of IBD and neuro-inflammation as well as in the human adenocarcinoma cell line DLD-1.

## Discussion

The aim of the presented study was to evaluate the therapeutic potential of the fungal secondary metabolites dehydrocurvularin and galiellalactone, whose mechanism of action is quite different from glucocorticoids, for the potential treatment of intestinal inflammation. They were tested in a recently developed organ perfusion model (Schreiber et al., 2014), combined with additional cell culture experiments using ENS derived primary cells as well as the DLD-1 cell line, which is derived from colonic epithelial cells. In our perfusion model and in the cellular test systems, the administration of a pro-inflammatory cytokine mix (10 ng/ml TNF-α, 10 ng/ml IFN-γ, 5 ng/ml IL-1β) through the mesenteric artery caused a significant up-regulation of inflammatory marker genes, similar to those seen in IBD (Sanchez-Muñoz et al., 2008; Strober and Fuss, 2011; Christophi et al., 2012), so that a realistic induction and treatment of inflammation could be simulated. Additional experiments on intestinal cell lines and isolated myenteric plexus were performed to demonstrate the immediate impact on ENS and enterocytes. Especially with respect to the characteristic symptoms such as abdominal pain and motility disorders, the contribution of the ENS to IBD is also quite obvious. The ENS is located in intramural plexus in which neuroinflammatory processes during IBD take place (Lakhan and Kirchgessner, 2010; Margolis et al., 2011; Demir et al., 2013).

Dehydrocurvulain and galiellalctone were isolated by fermentation of the original producer strains as stated in the Material and Methods section by our lab. These compounds were found in course of a screening for anti-inflammatory compounds and initially characterized in cell culture models as cited in the references. To the best of our knowledge this is the first report on the effect of both compounds in a model of IBD and since these compounds are investigational new drugs, no data on pharmacokinetics are yet available. The aim of the present study was to show that concentrations used in the cell culture models indeed show anti-inflammatory activities in a more advanced model. In our previous study with galiellalactone in apo-E deficient mice, we did not observed any acute toxicity of galiellalactone during a 6 weeks treatment (i.p. injection) of 10 mg/kg of the compound (unpublished data).

### Intestinal perfusion

In the luminal and mesenterially perfused model, oxygen supply can be guaranteed through the cannulated mesenteric superior artery as well as through luminal perfusion and serosal superfusion with oxygenated media. Furthermore, the vasal application of pharmacologically active compounds can easily be realized.

Potential tissue penetration or the route of application of pharmacological compounds can alter their effectiveness. Therefore, parenteral application through the mesenteric superior artery was chosen for this study. In comparison to live animal models, where the route of application is a key factor, perfusion models allow a higher reproducibility due to the fact that various disruptive factors can be excluded. The perfusion model is less time consuming, less expensive and in particular affords much lower amounts of test compounds. As a conclusion, it is ideal for experimental drugs of which only small amounts are available. Compared to cell culture, perfusion models offer a much higher physiological complexity which is closer to the *in vivo* situation.

In comparison to alternative methods for live animal tests, whose development begun during the last two decades, numerous live animal models for IBD are available. At least 66 animal models have been developed (Mizoguchi, 2011), among them 50 established in mice (Jones-Hall and Grisham, 2014). None of these models can fully reproduce the human immunopathology of Crohn's disease or ulcerative colitis and every model offers only specific advantages and similarities (Jones-Hall and Grisham, 2014). Keeping those facts concerning animal models in mind, a perfusion model using cytokines as a pro-inflammatory stimulus offers several advantages. As a general, unspecific inflammation model, it offers central mechanisms of inflammation and does not just depict single aspects of a specific disease. Thus, anti-inflammatory compounds, that inhibit pro-inflammatory pathways, can easily be characterized.

### The ENS and intestinal inflammation

The ENS is responsible for intestinal movements in the perfusion model. It is the intrinsic innervation of the gastrointestinal tract. Two different cell types are part of the ENS: neurons and glial cells which are arranged in two intramural plexus. Besides the coordination of intestinal motility, the ENS has a broad range of additional functions (Sasselli et al., 2012). Due to its central function during intestinal inflammation, the ENS has come into focus as an important target for anti-inflammatory drugs. Based on an intense neuro-immune interaction, the ENS takes an active part in intestinal immunology (von Boyen et al., 2004; Fruhwald et al., 2006; Lakhan and Kirchgessner, 2010; Margolis et al., 2011; Demir et al., 2013). In case of faulty regulation, different clinical outcomes can arise on the basis of ENS problems. In the case of IBD, the ENS takes a key role because it is directly or indirectly responsible for symptoms such as diarrhea, hypersensitivity, pain, motility disorders, and other digestive problems (Lakhan and Kirchgessner, 2010; Margolis et al., 2011; Demir et al., 2013). The ENS is a central player for gut function and also a specific target in IBD (von Boyen et al., 2004; Fruhwald et al., 2006; Lakhan and Kirchgessner, 2010; Margolis et al., 2011; Sasselli et al., 2012; Demir et al., 2013). Recent studies show that the ENS also plays a crucial role in protecting the intestine from pathogens and inflammation (Sharkey and Savidge, 2014) while different sources state morphological and physiological changes in the ENS (Lomax et al., 2005; Vasina et al., 2006). These changes are directly associated with motility disorders, diarrhea and abdominal pain. Between enteric glial cells and lymphocytes, functional interactions have already been documented in the past (Hirata et al., 1986; Koretz et al., 1987; Geboes et al., 1992; Ruhl et al., 2001).

The presented study demonstrates that the ENS responds to a cytokine stimulus similar to the one in IBD (Sanchez-Muñoz et al., 2008; Strober and Fuss, 2011; Christophi et al., 2012) with a massive secretion of pro-inflammatory cytokines and with an increase in pro-inflammatory gene expression. Pro-inflammatory reactions could be modulated with anti-inflammatory compounds. For enteric glial cells, iNOS expression was shown in culture within this study using an anti iNOS staining. Most studies with different IBD models showed positive effects of iNOS inhibitors on the disease (Cross and Wilson, 2003). Our test compounds are potent inhibitors of iNOS expression. Nitric oxide (NO) as a product of the iNOS is a component of the unspecific immune response. It is part of the defense of macrophages against different pathogens such as bacteria, virus, and fungi. It is also a central component in the defense against tumor cells (Keller et al., 1991; Liew and Cox, 1991; Bogdan, 2001; Malysheva et al., 2006; Nahrevanian, 2006). In the course of acute and chronic inflammatory diseases such as IBD, sepsis, rheumatoid arthritis and bronchial asthma, NO is produced in great quantities by the iNOS. As an effector molecule of immune cells, it enhances the inflammatory and immune response, but can also lead to cell and tissue damage.

The induction of nitric oxide production is a part of the acute intestinal antibacterial immune reaction. The iNOS is strongly involved in the initiation and maintenance of different forms of mucosal inflammation (Cross and Wilson, 2003; Kolios et al., 2004). Different studies have shown that iNOS expression, enzymatic NOS activity and NO production are increased in IBD patient's tissue. According to Cross, the amount of nitric oxide correlates with disease activity, especially in ulcerative colitis (Cross and Wilson, 2003). Avdagic showed that the serum concentration of NO in Crohn's disease and ulcerative colitis patients is significantly increased in comparison to the control group (Avdagic et al., 2013).

In myenteric plexus cultures, the anti iNOS staining showed that the protein is restricted to glial cells. The iNOS expression was inhibited by the test compounds galiellalactone and dehydrocurvularin. These results could be corroborated for both postnatal and adult myenteric plexus. The iNOS expression is mainly regulated by JAK/STAT and NF-κB pathways (Kleinert et al., 2003, 2004; Aktan, 2004). In accordance to that, the inhibition of NF-κB and STAT1 leads to a decrease in iNOS expression. The effects of dehydrocurvularin and galiellalactone as observed in the perfusion model as well as in the cell culture are based on these effects. Due to the fact that the iNOS gene does not contain a glucocorticoid responsive element (GRE), effects of dexamethasone must be indirect (Kleinert et al., 1996). The observed iNOS suppressing effects of dexamethasone are possibly based on other mechanisms, such as the inhibition of NF-κB or the inhibition of the histone deacetylase (Matsumura et al., 2001; Hamalainen et al., 2007).

### Discussion of the results

Dehydrocurvularin and galiellalactone have proven to be effective on inflammatory processes in the ENS and on the DLD-1 epithelial cell line, as well as in the mesenterially perfused intestine. In the latter, the quality of the tissue was tested after long periods of perfusion (5 h) with neglectable damage. Shorter periods of perfusion time seem to interfere even less with the tissue quality (Lautenschlager et al., 2010). Motility could be observed in all perfusion experiments. In an intestinal perfusion model, complex interactions between different tissues and the immune system can be simulated. Especially the mucosal immune system, which is completely functional in perfused segments, is a key factor and exhibits a crucial role on the efficacy of pharmaceutical treatment.

The fungal secondary metabolites galiellalactone and dehydrocurvularin were identified and characterized in previous studies as potent anti-inflammatory compounds and inhibitors of NF-κB, TGF-β, and JAK/STAT pathways (Weidler et al., 2000; Hellsten et al., 2011; Rudolph et al., 2012). Anti-inflammatory effects of galiellalactone and dehydrocurvularin on intestinal tissue could be validated in the course of this study. In experiments with ENS cells in the qRT-PCR and Multiplex ELISA, similar effects could be observed for both compounds at 10.3 μM (Figure 3). On perfused tissue, galiellalactone (20.6 μM) was more effective than dehydrocurvularin (20.7 μM) on mRNA level (Figure 2C) and in Multiplex ELISA experiments (Figures 2A,B) for most readouts. Dehydrocurvularin seems to be less effective on intestinal tissue which could be due to a better tissue availability of galiellalactone. Both anti-inflammatory compounds showed a better efficacy on ENS cells and perfused tissue than the control compound dexamethasone.

The observed reductions of critical mediators (e.g., iNOS, cytokines, and chemokines) of inflammation indicate the potency of the fungal metabolites dehydrocurvularin and galiellalactone as potential new drugs for the treatment of neuro-inflammatory processes. In addition, we provided evidence that the inflammatory and anti-inflammatory response of the adult and postnatal ENS *in vitro* are similar due to the fact, that iNOS protein expression is decreased in both cell types by the fungal metabolites.

Dehydrocurvularin and galiellalactone significantly reduced the expression of pro-inflammatory mediators such as cytokines (e.g., IL-1β, IL-6), chemokines (e.g., CXCL10, IL-8), and enzymes (e.g., iNOS, COX-2) in ENS- as well as DLD-1 cells and also in perfused intestinal tissue as shown by qRT-PCR. For some transcripts, this decrease in gene expression could also be verified on protein level.

The intestinal perfusion model used in this study is a short time model. Crohn's disease and ulcerative colitis develop over longer periods of time with a strong involvement of the adaptive immune system. Therefore, this model cannot depict all aspects of Crohn's disease or ulcerative colitis. The central feature shared by both diseases is tissue inflammation caused by acute and later chronic cytokine release. Crohn's disease is associated with a Th_1_ and Th_17_ cytokine profile, while ulcerative colitis is associated with a Th_2_ cytokine profile (Strober and Fuss, 2011). Both diseases are associated with the activity of different T-cell populations. Their disease specific cytokines lead to the secretion of secondary cytokines by different types of cells, the so called upstream facilitators and downstream mediators. Among them are TNF-α and IL-1β which were used as a stimulus in our model. Those cytokines are not disease specific, but rather are a general sign of inflammation, which is associated with both diseases (Strober and Fuss, 2011). Due to the fact that our model is a short time model, we tried to imitate central aspects of the inflammatory process, which both diseases share. As a consequence, the model shown can be considered to be similar to both forms of IBD. The results of this study justify further testing of the anti-inflammatory compounds in a chronic animal model of IBD.

### Summary of the discussion

Different animal models of IBD are available at the moment. The main problem of these models is their resistance to drugs which are firmly established in human medicine such as 5-ASA and glucocorticoids (Danese, 2011). Therefore, a general intestinal inflammation model such as the perfusion model, which depends on the application of pro-inflammatory cytokines, can be favorable for drug testing. Similar to the human pathological situation, inflammatory symptoms depend largely on different key cytokines like TNF-α.

Currently used anti-inflammatory therapeutics are often based on glucocorticoids. Anti-inflammatory therapy based on glucocorticoids can provoke serious side effects based on their mechanism of action. Side effects based on glucocorticoid receptor interaction can be ruled out for our novel anti-inflammatory drugs due to the fact that their mechanism of action is entirely different. Anti-inflammatory compounds like dehydrocurvularin and galiellalactone, which cover a broad range of activity, could also provide a useful alternative to more and more specific biologicals. For a combination therapy as e.g., established for infliximab and azathioprin, novel anti-inflammatory compounds could be interesting (Colombel et al., 2010). The mechanism of action of the novel anti-inflammatory compounds, dehydrocurvularin and galiellalactone is different form glucocorticoids as well as NSAIDs and modern biologicals. Pro-inflammatory transcription factors like STAT1 and NF-κB as well as the TGF-β pathway are inhibited (Rudolph et al., 2012). Together with novel testing systems like the presented luminal and vasal perfused intestine model, novel anti-inflammatory compounds can help to reduce side effects like motility disturbance and to predict them already during drug development and thereby accelerate the complete process. In general, we could successfully demonstrate that new anti-inflammatory compounds with strong effects are available.

## Author contributions

Participated in research design: KS, GE, and DS; conducted experiments: DS, SF, JC, MS, LM, MW, and RL; contributed new reagents or analytic tools: GE; performed data analysis: DS, SF, MS, and LM; wrote or contributed to the writing of the manuscript: GE, KS, MK, and DS.

### Conflict of interest statement

The authors declare that the research was conducted in the absence of any commercial or financial relationships that could be construed as a potential conflict of interest.

## Supplementary material

Supplementary Figure 1 Surgical isolation of the luminal and vasal perfused intestine **(A)** and perfusion setup **(B)**. **(A)** Extraction of luminal and vasal perfused mouse intestinal segments. After anesthesia, the abdominal cavity was opened. Parts of the intestine, which were not intended for perfusion, were ligated and resected. In the last step, heparin was injected into the heart, the mesenteric superior artery was cannulated and the segment was put into a perfusion bath. **(B)** Perfusion setup used in this study. The intestinal segment was held in the perfusion bath by a custom designed fastener. The mesenteric root was held upright in the bath. The superfusion as well as luminal and vasal perfusion medium were oxygenated, temperated and pumped by different systems as described in the Materials and Methods section.

Supplementary Figure 2 Example picture of the intestinal motility during luminal and vasal combined perfusion. The intestinal segment was resected from the jejunum and perfused for 5 h with vehicle only (uninduced control). Intestinal motility was evaluated and graphically depicted in heatmap images as described in the Materials and Methods Section-Intestinal perfusion and perfusion system. The x-axis shows the timeline while the y-axis shows the intestine. Its movement is color coded. When the intestinal border moves downward, the heatmap becomes more red/violet, when it moves upward, the heatmap becomes more blue. The annotations of the axes of all individual heatmaps are identical.

Supplementary Figure 3 Overview staining of the postnatal ENS single cell culture **(A–C)**: Neurons (green, βIII-Tubulin), glial cells (red, GFAP) and cell nuclei (blue, DAPI). iNOS expression in the CM-treated culture **(D–F)**: Neurons (green, βIII-Tubulin), iNOS positive cells (red, iNOS) and cell nuclei (blue, DAPI). Cells were cultured as described in the Materials and Methods section. 5,000 cells/coverslip.

Supplementary Figure 4 Multiplex ELISA of culture supernatants from adult ENS networks after treatment with the anti-inflammatory compounds galiellalactone and dehydrocurvularin. Cells were pretreated for 1 h with the indicated concentrations of test compounds prior to stimulation with CM for 24 h. The data are presented in comparison to untreated control cells (±*SD*). The untreated control cells correspond to 100%. *n* = 3 for the test substances galiellalactone and dehydrocurvularin, *n* = 2 for the positive control dexamethasone. The supplementary data for IL-22 is shown in this figure in comparison to the data from Figure 3 (IL-22 *n* = 2). ^*^*p* < 0.05; ^**^*p* < 0.01; ^***^*p* < 0.001 treated cells vs. cytokine induced cells.

## Abbreviations

ApoE: apolipoprotein E
BSA: bovine serum albumin
CM: cytokine mix, human cytokines for human cell lines, mouse cytokines for murine cells and tissue
CNS: central nervous system
COX-2: cyclooxygenase-2
Deh: dehydrocurvularin
Dex: dexamethasone
ECM: extracellular matrix
ELISA: enzyme-linked immunosorbent assay
ENS: enteric nervous system
ERK: extracellular signal-regulated kinases
EtOH: ethanol (p.a.)
FCS: fetal calf serum
Fps: frames per second
Gal: galiellalactone
GAPDH: glyceraldehyde-3-phosphate dehydrogenase
h: hours
HMDS: Bis(trimethylsilyl)amine
HPLC: high-performance liquid chromatography
IBD: inflammatory bowel diseases
ind.: induced
iNOS: inducible nitric oxide synthase
JAK: janus kinase
JNK: c-Jun N-terminal kinases
MAPK: mitogen-activated protein kinases
NF-κB: nuclear factor kappa-light-chain-enhancer of activated B cells
NO: nitric oxide
NSAIDs: Nonsteroidal anti-inflammatory drugs
PBS: phosphate buffered saline
qRT-PCR: quantitative real-time polymerase chain reaction
RNA: ribonucleic acid
SD: standard deviation
SOD: superoxide dismutase
STATs: Signal Transducers and Activators of Transcription
TNBS: trinitrobenzenesulfonic acid
vs.: versus.
