# Supplementary figures and images for: Anti-inflammatory Effects of Fungal Metabolites in Mouse Intestine as Revealed by In vitro Models
Source: Front Physiol. 2017 Aug 7;8:566. doi: 10.3389/fphys.2017.00566 (PMC5545603; doi:10.3389/fphys.2017.00566)

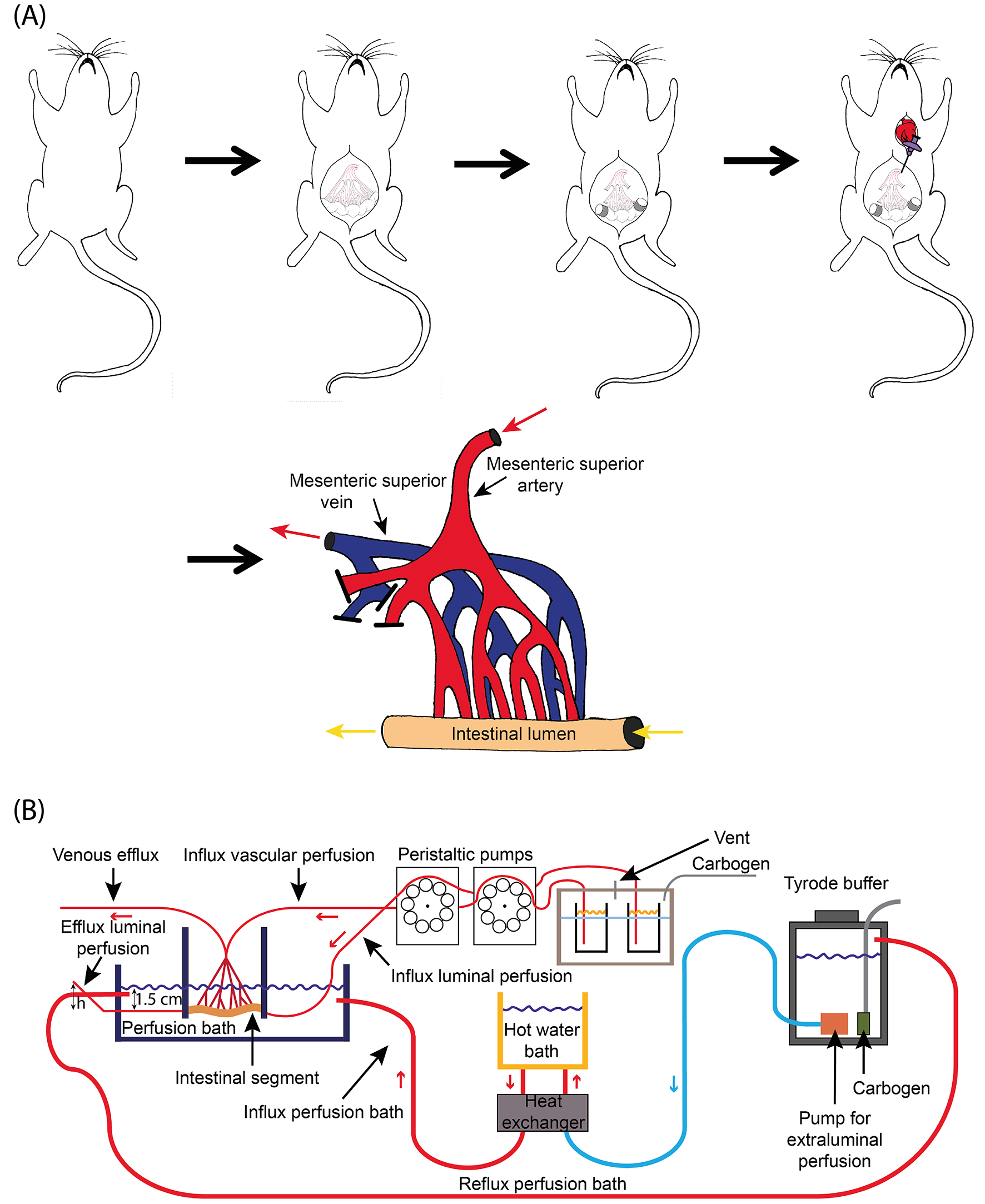

Supplement: Supplementary Figure 1 — Surgical isolation of the luminal and vasal perfused intestine (A) and perfusion setup (B). (A) Extraction of luminal and vasal perfused mouse intestinal segments. After anesthesia, the abdominal cavity was opened. Parts of the intestine, which were not intended for perfusion, were ligated and resected. In the last step, heparin was injected into the heart, the mesenteric superior artery was cannulated and the segment was put into a perfusion bath. (B) Perfusion setup used in this study. The intestinal segment was held in the perfusion bath by a custom designed fastener. The mesenteric root was held upright in the bath. The superfusion as well as luminal and vasal perfusion medium were oxygenated, temperated and pumped by different systems as described in the Materials and Methods section. [file Image1.TIF]

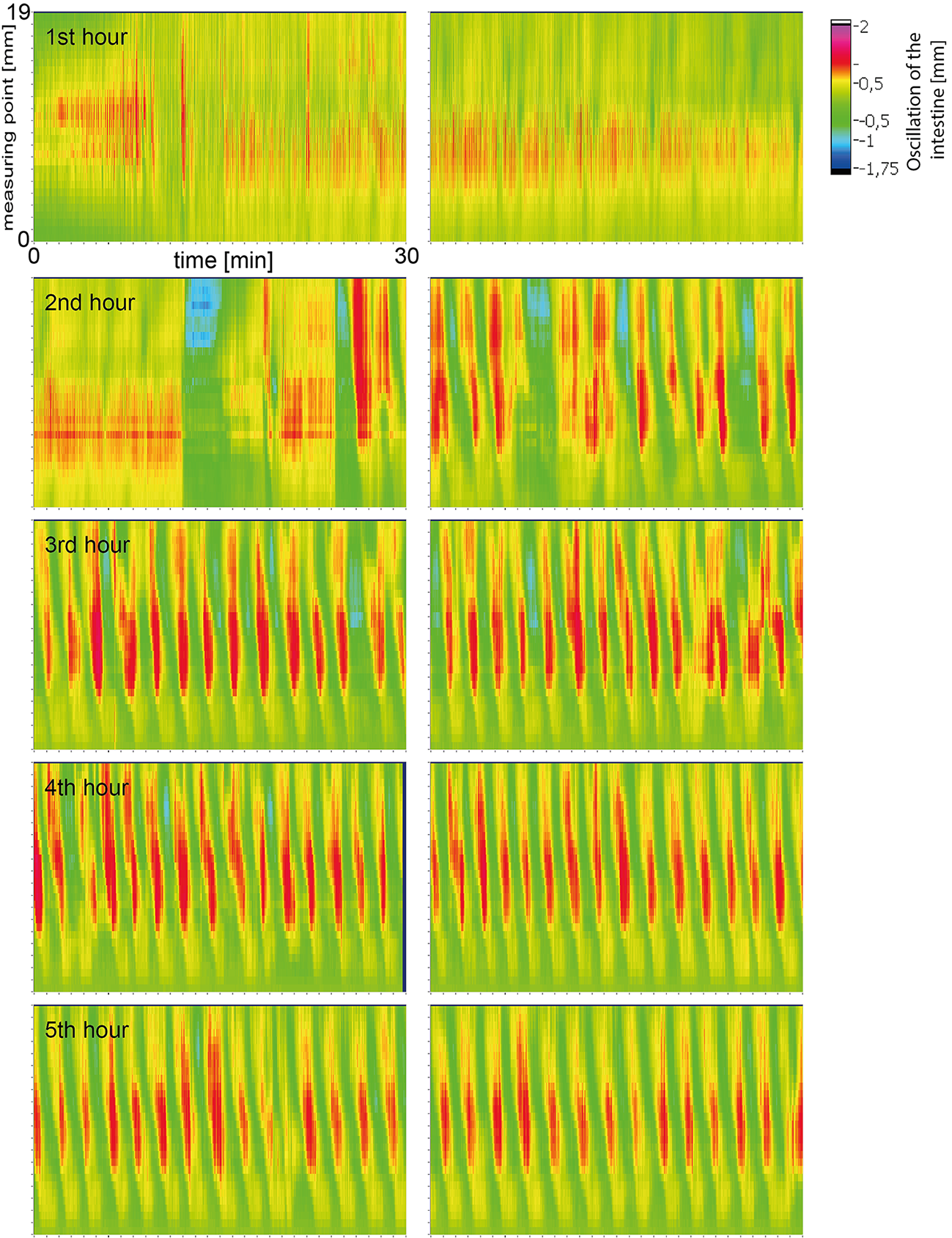

Supplement: Supplementary Figure 2 — Example picture of the intestinal motility during luminal and vasal combined perfusion. The intestinal segment was resected from the jejunum and perfused for 5 h with vehicle only (uninduced control). Intestinal motility was evaluated and graphically depicted in heatmap images as described in the Materials and Methods Section-Intestinal perfusion and perfusion system. The x-axis shows the timeline while the y-axis shows the intestine. Its movement is color coded. When the intestinal border moves downward, the heatmap becomes more red/violet, when it moves upward, the heatmap becomes more blue. The annotations of the axes of all individual heatmaps are identical. [file Image2.TIF]

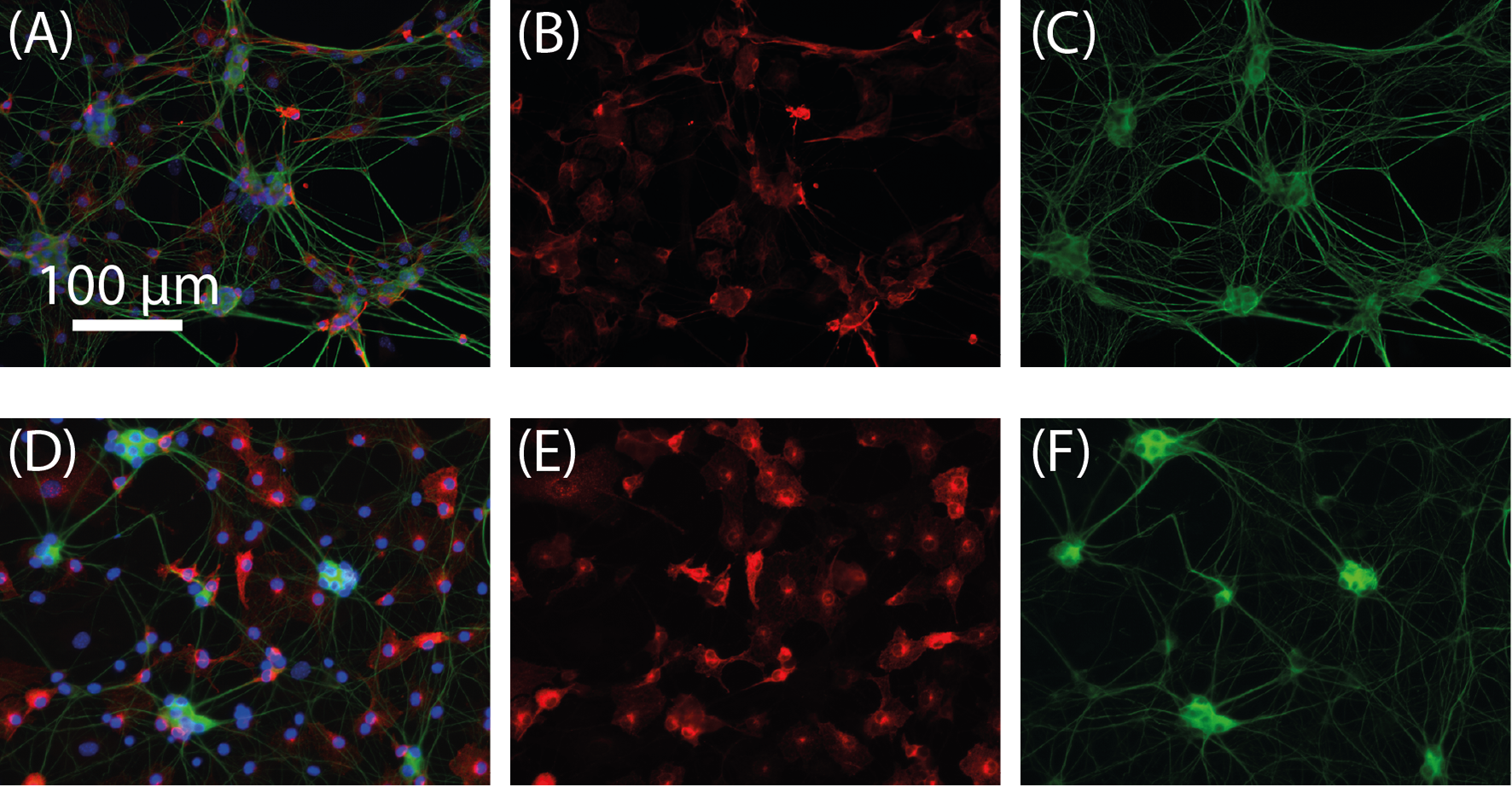

Supplement: Supplementary Figure 3 — Overview staining of the postnatal ENS single cell culture (A–C): Neurons (green, βIII-Tubulin), glial cells (red, GFAP) and cell nuclei (blue, DAPI). iNOS expression in the CM-treated culture (D–F): Neurons (green, βIII-Tubulin), iNOS positive cells (red, iNOS) and cell nuclei (blue, DAPI). Cells were cultured as described in the Materials and Methods section. 5,000 cells/coverslip. [file Image3.TIF]

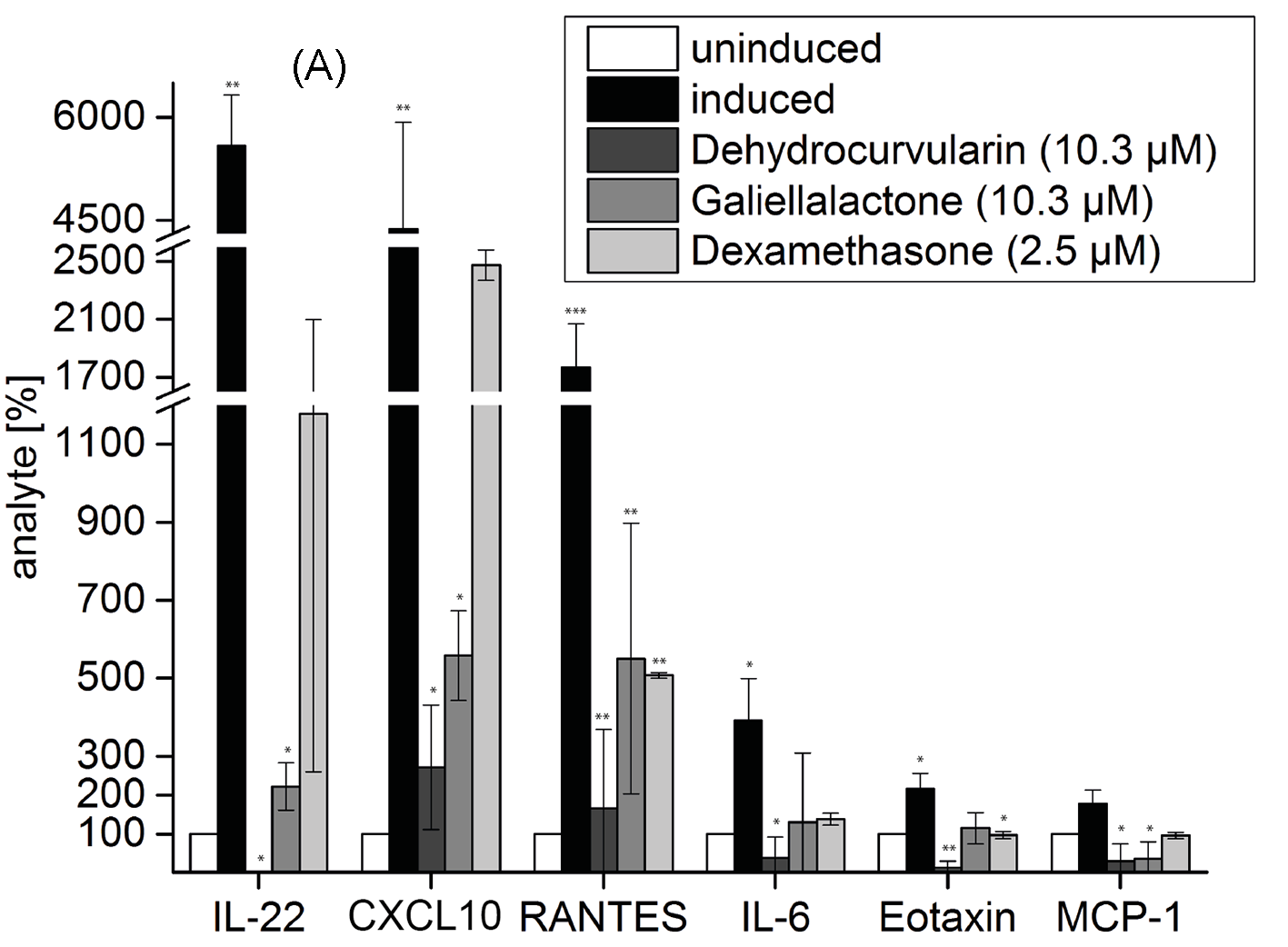

Supplement: Supplementary Figure 4 — Multiplex ELISA of culture supernatants from adult ENS networks after treatment with the anti-inflammatory compounds galiellalactone and dehydrocurvularin. Cells were pretreated for 1 h with the indicated concentrations of test compounds prior to stimulation with CM for 24 h. The data are presented in comparison to untreated control cells (±SD). The untreated control cells correspond to 100%. n = 3 for the test substances galiellalactone and dehydrocurvularin, n = 2 for the positive control dexamethasone. The supplementary data for IL-22 is shown in this figure in comparison to the data from Figure 3 (IL-22 n = 2). *p < 0.05; **p < 0.01; ***p < 0.001 treated cells vs. cytokine induced cells. [file Image4.TIF]
